# Surviving the Storm: Insights Into the Pericardial Injuries and Roller Coaster of Multisystem Trauma in Bomb‐Blast Victims

**DOI:** 10.1002/ccr3.70314

**Published:** 2025-03-14

**Authors:** Muhammad Nadeem Ahmad, Shahzeb Ali, Muhammad Ahmed, Naila Nadeem, Mallick Muhammad Zohaib Uddin, Hatem Eltaly, Muhammad Owais Rao, Faheemullah Khan, Uffan Zafar

**Affiliations:** ^1^ The Aga Khan University Hospital Karachi Pakistan; ^2^ Quaid‐e‐Azam Medical College Bahawalpur Punjab Pakistan; ^3^ Cleveland Clinic Main Campus Hospital Cleveland Ohio USA

**Keywords:** bomb blast, CT scan, multidisciplinary approach, pericardial injury, trauma

## Abstract

Pericardial injuries due to penetrating trauma such as bomb‐blast shrapnel are rare but are associated with significant mortality. Timely diagnosis and a multidisciplinary approach are essential for effective management. This case series, comprising two cases, emphasizes the need for rapid, appropriate imaging and multidisciplinary care in the management of all associated injuries in these complex polytrauma patients. It also underscores the need for a high index of suspicion despite negative clinical features of pericardial injuries for timely performing CT scans and adaptive treatment strategies, including life‐saving surgical intervention.

**Summary**

- Pericardial injuries from bomb blasts are rare but life‐threatening.
- Early diagnosis through contrast‐enhanced CT scans and a multidisciplinary approach are crucial for effective management in such complex trauma cases.
- Timely imaging, surgical intervention, and postoperative monitoring are key to reducing mortality and morbidity in these patients.

## 1 Introduction

Blast injuries, particularly in work‐related or combat scenarios, can result in a wide range of trauma, including primary (due to the blast wave), secondary (due to shrapnel and debris), tertiary (due to displacement of the victim), and quaternary injuries (due to burns or inhalation of toxic substances) [1]. Among these, secondary blast injuries, such as those caused by shrapnel, are particularly relevant to pericardial injuries. The shrapnel can penetrate the thoracic cavity, leading to pericardial and cardiac damage, which, if not promptly diagnosed and managed, can be fatal. This case series highlights the challenges and management strategies for pericardial injuries in bomb‐blast victims, emphasizing the importance of a multidisciplinary approach.

Pericardial injuries due to penetrating trauma such as bomb‐blast shrapnel are rare but are associated with significant mortality. A previous study shows that out of 20,000 patients received in a Level 1 trauma center, only 59 patients had pericardial injury [2]. Another study calculated the mortality of all pericardial trauma at 28.4% [3]. The pericardium is a fibrous, sac‐like structure around the heart for its protection. However, due to its close proximity to other thoracic structures, it is prone to injury in thoracic trauma. Pericardial injury can result in cardiac tamponade, hemorrhagic shock, cardiac arrhythmia and arrest, and later on infection, and these complications can prove fatal [3]. Its management needs a timely and multidisciplinary approach including cardiothoracic surgeons, radiologists, and trauma surgeons.

This case series presents two such cases that highlight the significant challenges in the management of such injuries.

## 2 Case History

### 2.1 Case 1

A 30‐year‐old male presented to the ER with a bomb‐blast injury, resulting in injuries to his neck, chest, and right leg. His vitals were stable at the time of presentation, and GCS was 15/15. There was reduced air entry on the left side of the chest. The patient was transported to the hospital within 30 min of the blast and reached the radiology department within 45 min of arrival in the ER.

### 2.2 Case 2

A 17‐year‐old boy presented to the ER with a bomb‐blast injury in an unconscious state. He had injuries on his head and chest. His presenting vitals were stable, but GCS was low at 7/15. There was decreased air entry on chest auscultation and muffled heart sounds. The patient was transported to the hospital within 20 min of the blast and reached the radiology department within 30 min of arrival in the ER.

## 3 Differential Diagnosis, Investigations, and Treatment

### 3.1 Case 1

**FIGURE 1:**
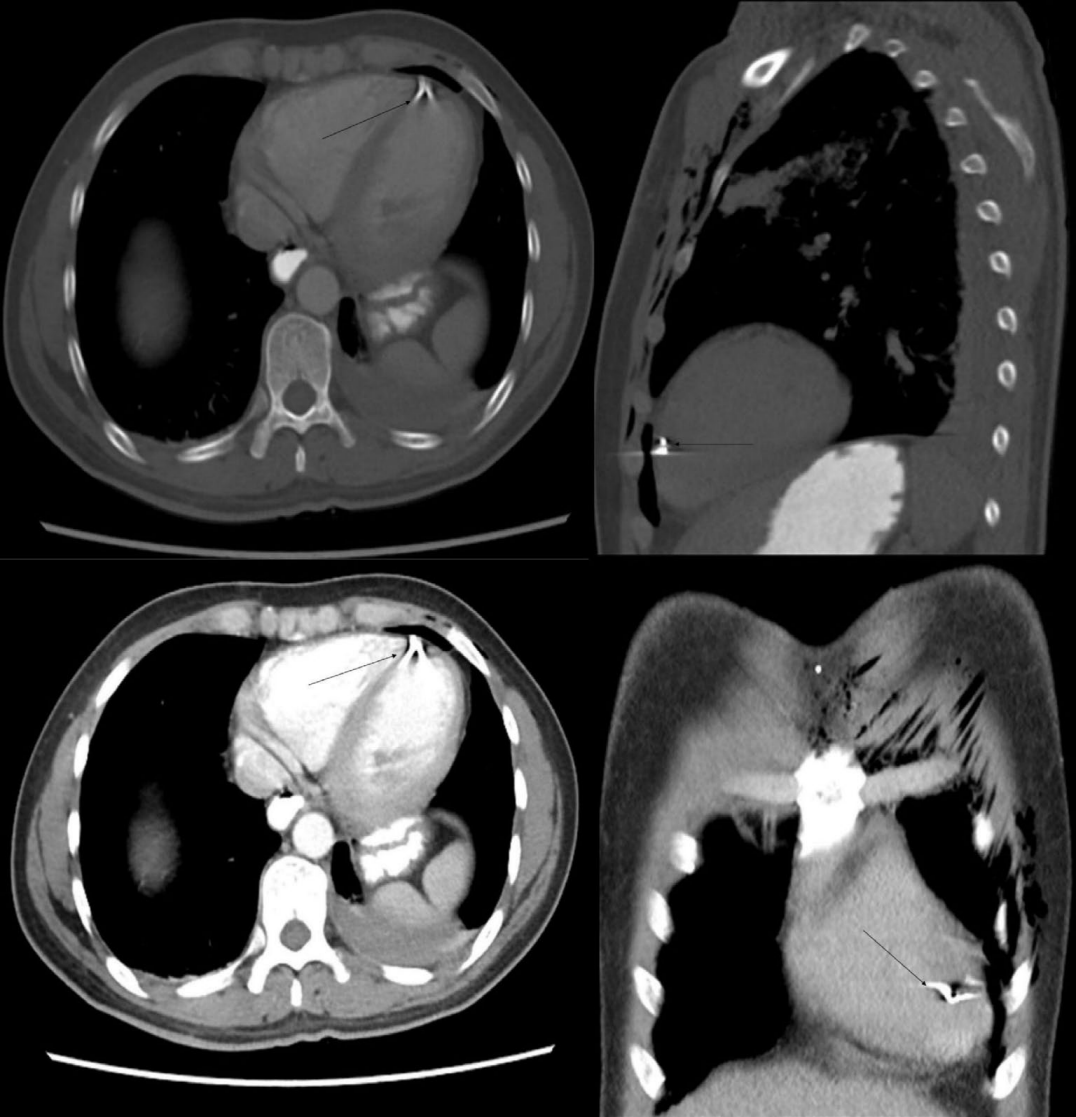
Contrast‐enhanced CT chest shows metallic density shrapnel (arrows) near the cardiac apex in the pericardium. Extensive subcutaneous emphysema in the anterior chest wall and left‐sided effusion (Case 1).

**FIGURE 2:**
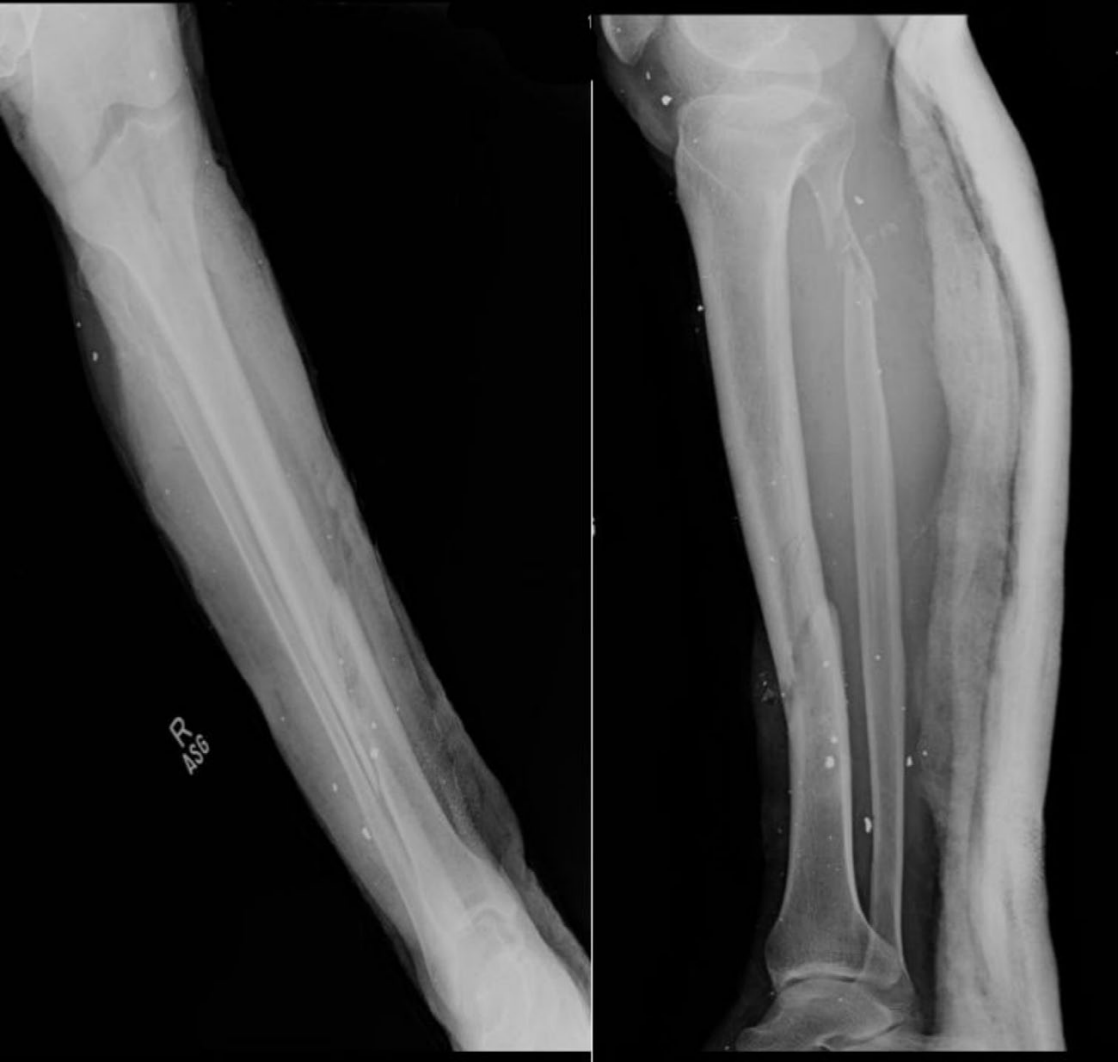
Radiograph of the right lower extremity showing a proximal fibular neck comminuted fracture and oblique tibial shaft fractures, along with tiny metallic densities in soft tissues (shrapnel) (Case 1).

The patient underwent CT of the neck and chest, and x‐rays of both legs in the ER. CT of the neck showed an undisplaced fracture of the left thyroid lamina with prelaryngeal soft tissue edema, while CT of the chest showed left‐sided upper lung lobe contusion and mild hemothorax. There was also significant soft tissue emphysema along the left chest wall, and a metallic shrapnel embedded in the pericardium at the cardiac apex (Figure 1). His right leg x‐rays showed an oblique fracture of the distal shaft of the right tibia along with a comminuted fracture of the proximal shaft of the fibula (Figure 2).

**FIGURE 3:**
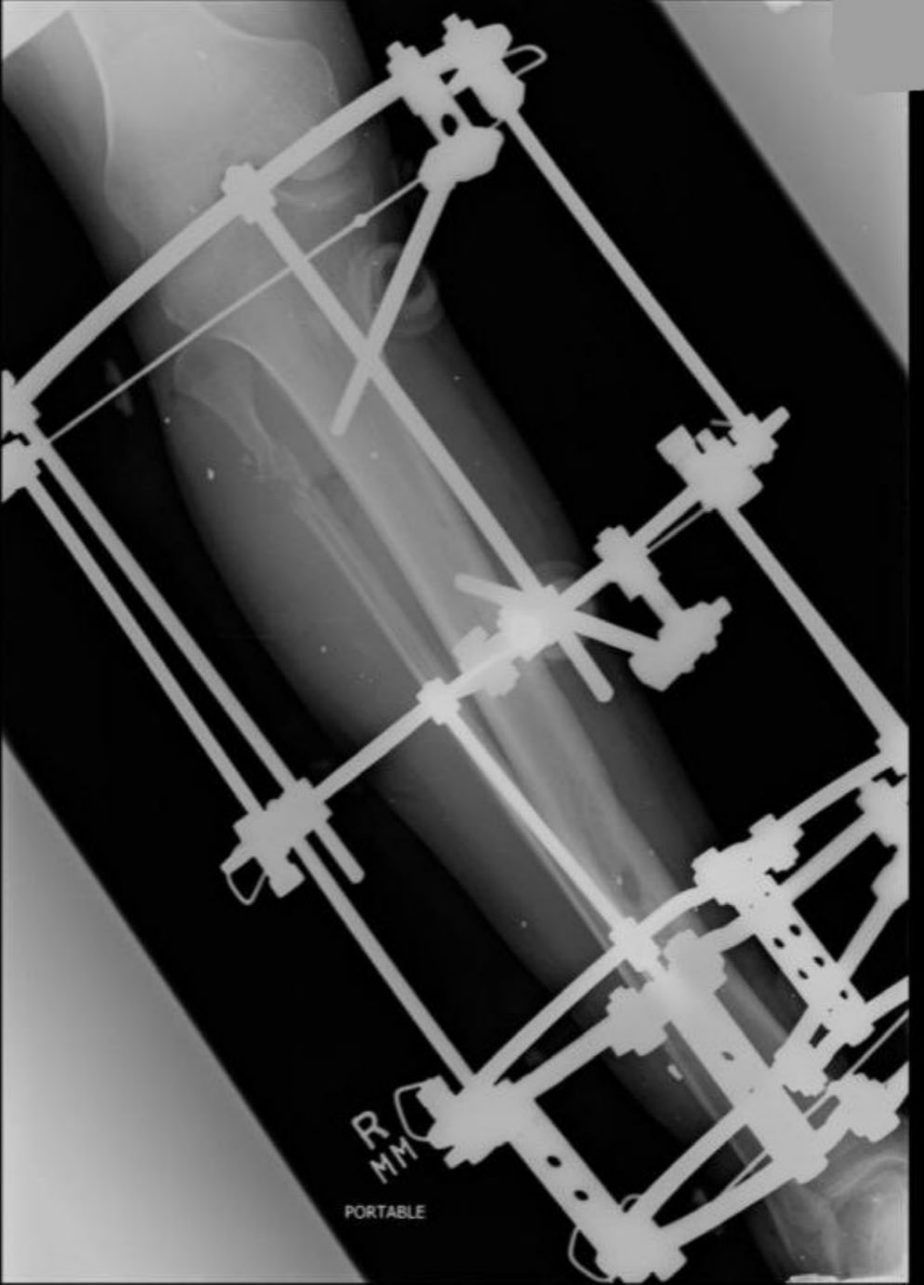
Radiograph of the right lower extremity showing status post Ilizarov fixation for tibial fracture (Case 1).

ENT ruled out the need for any urgent intervention because no great vessel was breached in the neck region and the airway was intact. The orthopedic team applied a backslab for initial stabilization of the tibia and fibula fracture. The patient was shifted to the OR for pericardial exploration and removal of the foreign body. However, cardiothoracic surgeons could not identify any foreign body in the pericardium despite its extensive exploration. Also, there was no pericardial effusion or contusion, laceration, or wound over the heart surface, so it was presumed that the foreign body was extrapericardial. He also underwent Ilizarov fixation of his right tibia after heart surgery (Figure 3).

### 3.2 Case 2

**FIGURE 4:**
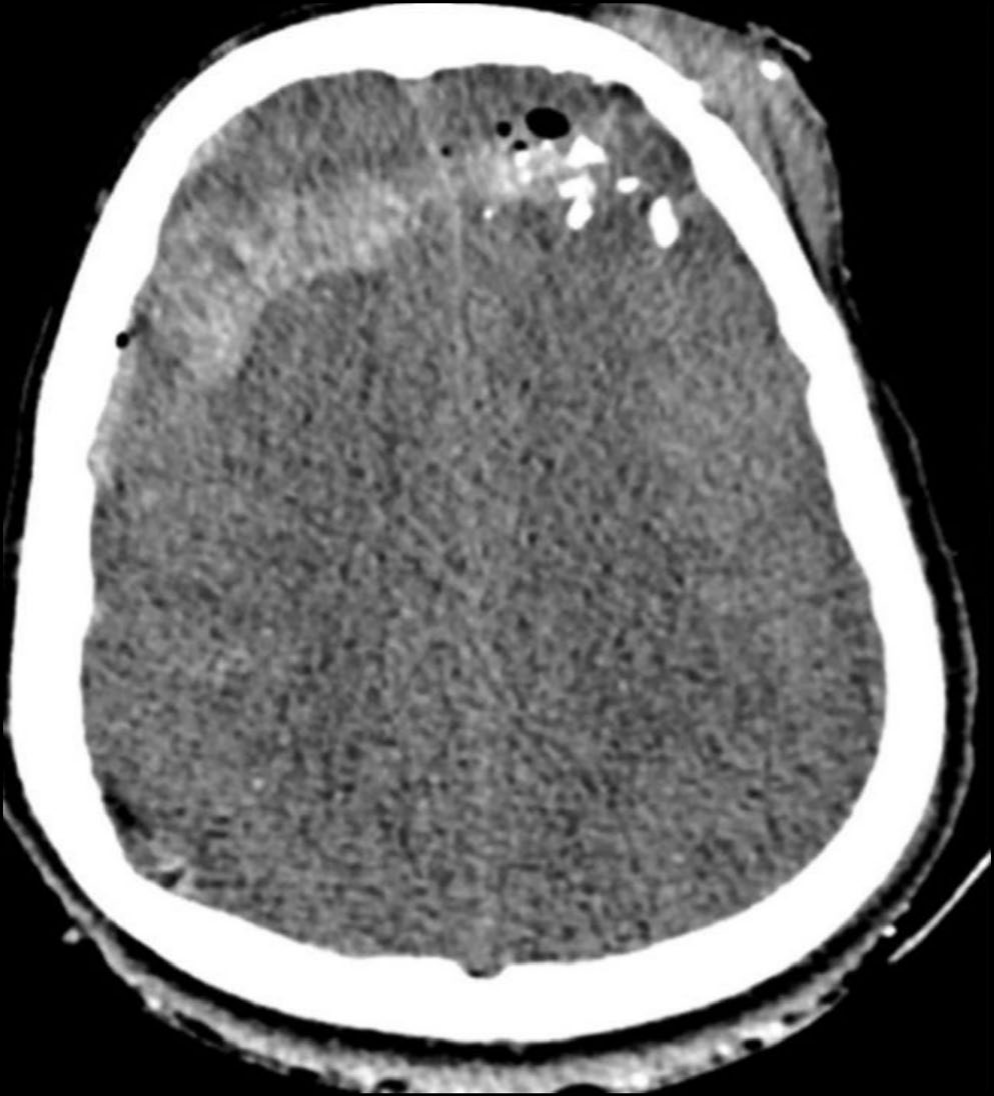
Initial CT showing intraparenchymal hemorrhage, pneumocephalus, diffuse cerebral edema, and scalp hematoma (Case 2).

**FIGURE 5:**
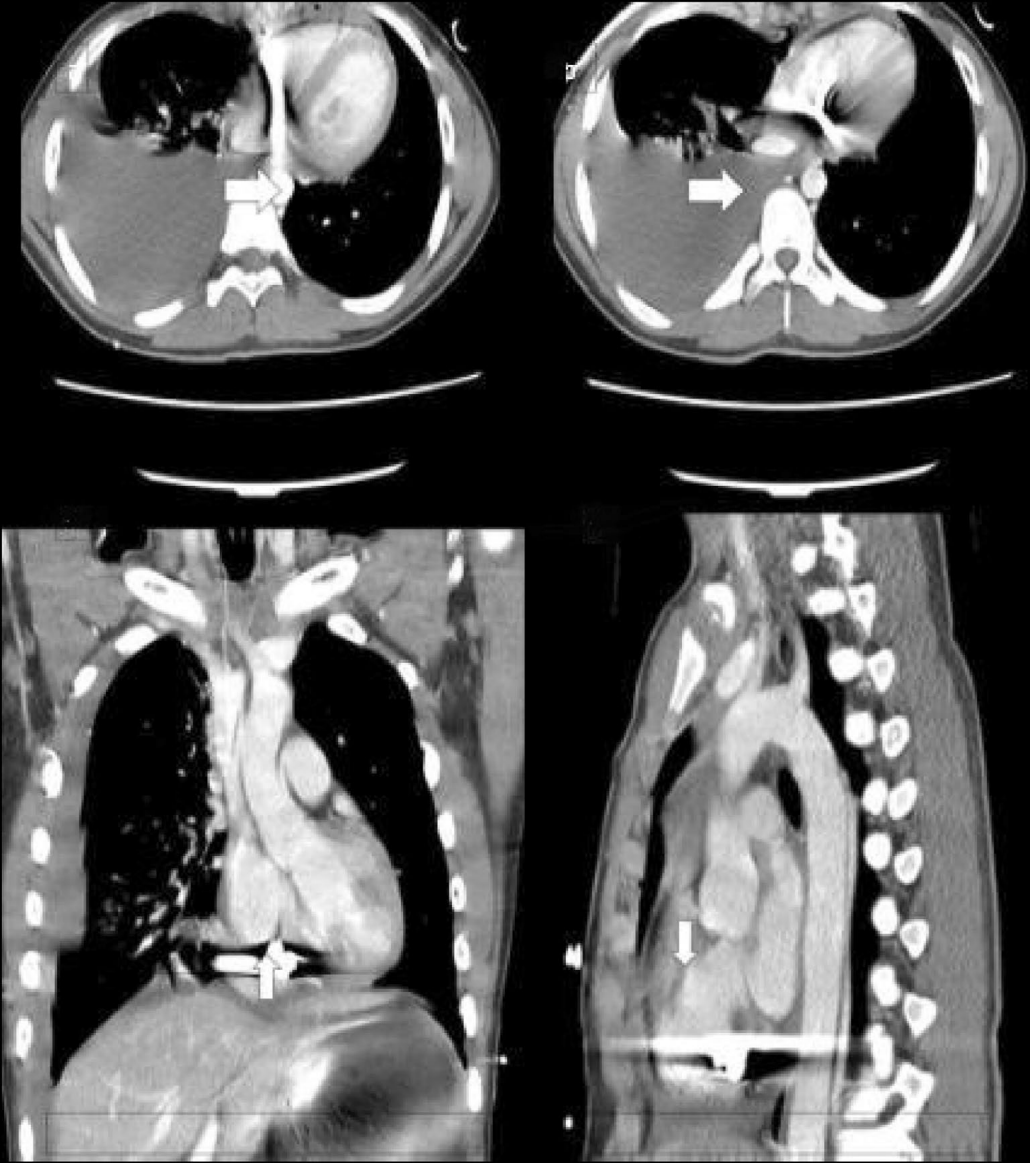
Contrast‐enhanced CT chest of the 17‐year‐old bomb‐blast victim: Multiplanar images show shrapnel fragments (arrows) within the pericardial space. Streak pericardial effusion and large right pleural effusion are seen (Case 2).

The patient immediately underwent CT scans of the head, neck, and chest, which showed a left frontal bone fracture, hemorrhagic contusions in the bilateral frontal and right parietal lobes with a metallic shrapnel embedded in the right parietal lobe (Figure 4). There were multiple metallic shrapnels in the chest wall and base of the heart with subcutaneous emphysema and gross hemothorax on the right side, underlying right lung contusion and atelectasis (Figure 5).

The patient was immediately rushed to the OR, where he first underwent median sternotomy and right ventricular repair. The shrapnel was seen impacted within the posterior wall of the heart, away from any vessel, and there was no oozing of blood through this foreign body. Therefore, the shrapnel was not removed. A chest tube was placed on the right side. Subsequently, the neurosurgery team took over the patient and performed bifrontal decompression and removal of the foreign body. Overall, it took almost 7 h of surgery, and the patient was transfused with seven units of packed RBCs and six units of FFPs and platelets intraoperatively.

The patient was kept in the ICU for 2 days and was then extubated, but his postoperative period was complicated by *Acinetobacter* and *Clostridium difficile* infections, which were managed by ID specialists. He was discharged in stable condition after 1 month.

## 4 Outcome and Follow‐Up

### 4.1 Case 1

The patient was kept in the ICU for 1 day and shifted out to the ward the next day. His postoperative management included morphine infusion for pain, incentive spirometry, and physiotherapy to mitigate postoperative complications. He was gradually allowed full weight‐bearing and was discharged after 5 days in stable condition. The patient had no late complications on 1‐year follow‐up.

### 4.2 Case 2

**FIGURE 6:**
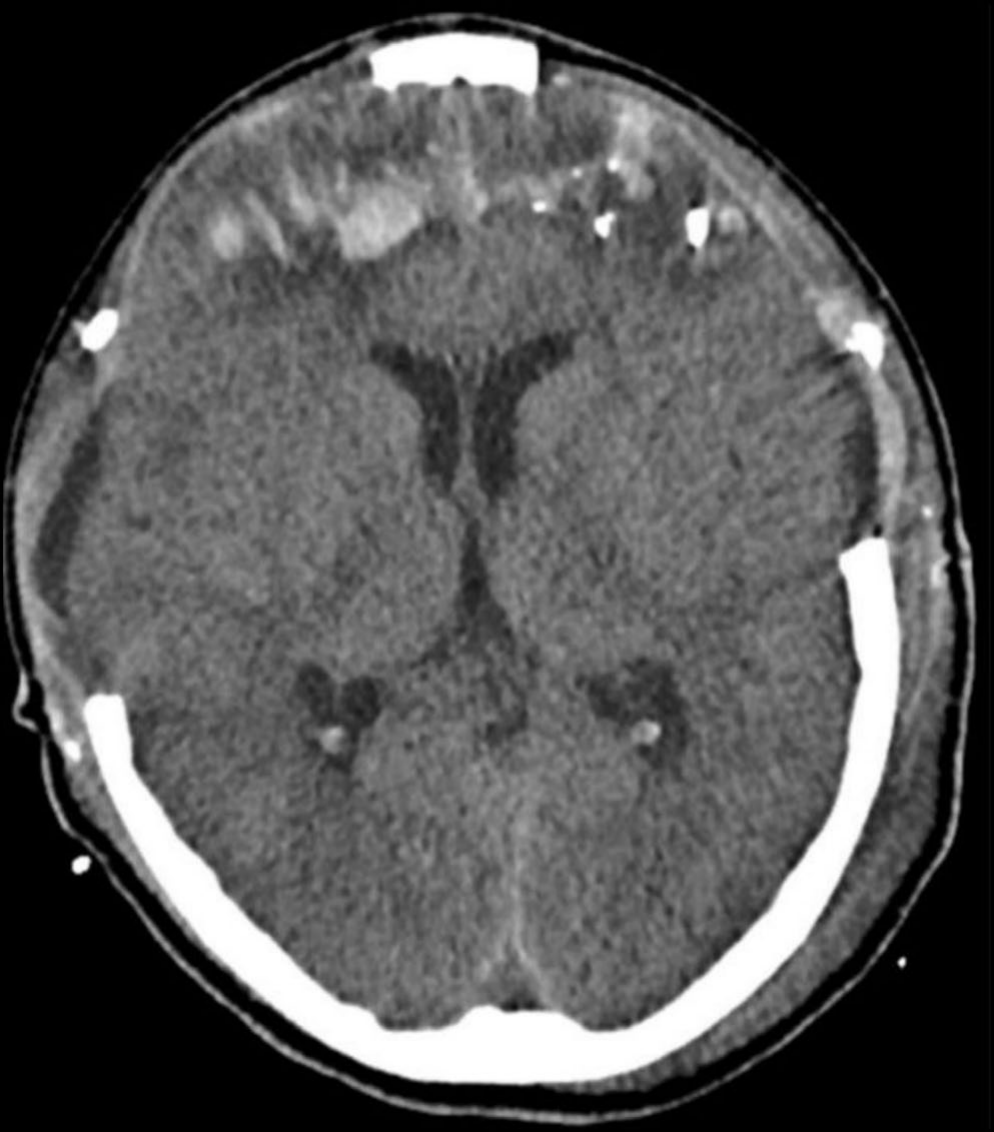
Follow‐up CT postdecompressive craniectomy shows improvement of cerebral edema, pneumocephalus, and hemorrhage (Case 2).

The patient was electively admitted for cranioplasty 2 months later. He underwent the procedure successfully, and there were no postoperative complications. A follow‐up CT postdecompressive craniectomy shows improvement in cerebral edema, pneumocephalus, and hemorrhage (Figure 6). The patient was discharged after 6 days. One‐year follow‐up showed no late complications in the patient.

## 5 Discussion

Pericardial injury is a rare occurrence in any trauma, and its timely diagnosis requires a high level of suspicion. CT scans and echocardiograms are invaluable in diagnosing pericardial injury and its complications. Timely imaging can be the difference between life and death, especially when clinical symptoms are not evident. A study of blunt and penetrating trauma patients shows that out of 11 patients with evidence of cardiac tamponade on imaging, only three had clinical features suggestive of it [4]. These imaging methods are crucial because they can detect small damage that may not be apparent during an initial clinical assessment. In both of our cases, patients were vitally stable throughout their admission period, although the presenting GCS of the second case was 7/15. In another case report in the literature, where the patient had self‐inflicted a stab wound to his left anterior chest, he presented in stable vitals, but soon after surgeons evacuated his left hemothorax, his BP dropped to 40/30 due to cardiac tamponade. He then underwent median sternotomy, opening of the pericardium, and evacuation of blood clots from it, which immediately stabilized his circulation [5].

Recent studies on penetrating cardiac injuries emphasize the importance of early imaging and surgical intervention. For instance, a study by Sessa et al. [6] highlights that contrast‐enhanced CT (CECT) scans are particularly useful in identifying pericardial and cardiac injuries, even in hemodynamically stable patients. This aligns with our findings, where CT scans played a pivotal role in diagnosing pericardial injuries in both cases.

In our second case, pericardial and right ventricular injury was easily diagnosed on a CT scan. This emphasizes the significance of rapid imaging in such situations and the necessity of maintaining a high index of suspicion, where timely intervention can be lifesaving. Most of the time, it becomes difficult to visualize the source of bleeding in pericardial injury on echocardiography. In that case, CECT can be used if the vitals of the patient permit. A case report of blunt trauma in the literature explains that initial echocardiography was not able to determine the source of pericardial blood that was causing cardiac tamponade as the underlying heart was not damaged. Only subsequent CECT was able to identify injury to the pericardiophrenic artery that was responsible for extravasation [7]. In another case report, an early chest x‐ray was normal without evidence of hemothorax, pneumothorax, or mediastinal widening and echocardiography was also inconclusive but suspicion of pericardial injury was high. The surgeons performed a surgical subxiphoid pericardial window that diagnosed hemopericardium [8]. This underscores the importance of a comprehensive diagnostic approach including proceeding directly to a CT scan or invasive procedures in such high‐risk cases.

Both cases highlight the significance of a multidisciplinary approach in managing this complex and life‐threatening injury and that timely intervention resulted in a good prognosis in each case. In such bomb‐blast cases, the teamwork of trauma surgeons, cardiothoracic surgeons, neurosurgeons, radiologists, and intensivists is crucial for the best patient outcomes. It is also crucial to prioritize and manage injuries based on their severity and urgency in the multisystem injuries of bomb‐blast victims. Pericardial injuries must be managed with priority because they can initially be asymptomatic but may quickly become life‐threatening. Early surgical intervention plays a crucial role in these scenarios, significantly reducing mortality and morbidity. It is also very significant to probe for other associated injuries that are often present in such scenarios. It is also very important to remove foreign bodies from the pericardium to prevent further damage and mitigate complications, including cardiac tamponade, hemorrhage, and infection. However, a risk–benefit analysis should be performed before the removal of these foreign bodies [9]. In our second case, the shrapnel was seen penetrating within the posterior cardiac wall. However, the shrapnel was not adjacent to any coronary vessel, sinoatrial node, or atrioventricular node. Therefore, the foreign body was not removed.

The second case had a postoperative complication of infection similar to another case report of pericardial injury in the literature, which improved with antibiotics [5]. It highlights the significance of proper postoperative care and monitoring to prevent complications, including lung atelectasis and infection. Our cases also demonstrated the significance of flexible and adaptive treatment methods. For example, the unexpected absence of a foreign body in the pericardium on surgical exploration in Case 1 necessitates a rethinking of our diagnostic assumptions, emphasizing the importance of ongoing review and revision of treatment regimens in such complex multisystem injuries.

Based on our experience and the literature, we propose the following workflow for managing pericardial injuries in bomb‐blast victims:

1. Initial assessment: Rapid primary survey (ABCs) and stabilization of the patient.
2. Imaging: Immediate CECT scan of the chest and other affected areas to identify pericardial and cardiac injuries.
3. Multidisciplinary approach: Involvement of cardiothoracic surgeons, trauma surgeons, and radiologists for timely intervention.
4. Surgical exploration: If pericardial injury is suspected, proceed with median sternotomy or subxiphoid pericardial window for definitive diagnosis and treatment.
5. Postoperative care: Close monitoring in the ICU for complications such as infection, arrhythmias, or tamponade.

## 6 Conclusion

This case series highlights the importance of a multidisciplinary approach and effective use of advanced diagnostics, and provides practical insights through detailed case management. Sharing such insights contributes to a better understanding and improved management of similar cases in the future. The study's small sample size limits the generalizability of the findings.

## Author Contributions

**Muhammad Nadeem Ahmad:** conceptualization, data curation, investigation, project administration, writing – original draft, writing – review and editing. **Shahzeb Ali:** writing – original draft, writing – review and editing. **Muhammad Ahmed:** conceptualization, data curation, supervision, validation. **Naila Nadeem:** conceptualization, data curation, investigation, supervision, validation, writing – original draft. **Mallick Muhammad Zohaib Uddin:** conceptualization, data curation. **Hatem Eltaly:** conceptualization, data curation, investigation, supervision, validation, writing – original draft, writing – review and editing. **Muhammad Owais Rao:** supervision, validation. **Faheemullah Khan:** conceptualization, supervision, validation. **Uffan Zafar:** conceptualization, data curation, investigation, project administration, writing – original draft, writing – review and editing.

## Ethics Statement

The authors have nothing to report.

## Consent

Written informed consent was obtained from the participants.

## Conflicts of Interest

The authors declare no conflicts of interest.

## Data Availability

The data supporting the findings of this study are available from the corresponding author upon reasonable request.
